# Comorbid Medical Conditions in Young Athletes: Considerations for Preparticipation Guidance During the COVID-19 Pandemic

**DOI:** 10.1177/1941738120939079

**Published:** 2020-06-24

**Authors:** Kimberly G. Harmon, Paul S. Pottinger, Aaron L. Baggish, Jonathan A. Drezner, Andrew M. Luks, Alexis A. Thompson, Sankar Swaminathan

**Affiliations:** † Departments of Family Medicine and Orthopaedics and Sports Medicine, University of Washington School of Medicine, Seattle, Washington; ‡ Department of Medicine, Division of Allergy and Infectious Diseases, University of Washington School of Medicine, Seattle, Washington; § Harvard Medical School, Cardiovascular Performance Program, Massachusetts General Hospital, Boston, Massachusetts; ‖ Department of Family Medicine, University of Washington School of Medicine, Seattle, Washington; ¶ Division of Pulmonary, Critical Care & Sleep Medicine, University of Washington School of Medicine, Seattle, Washington; # Hematology Section, Feinberg School of Medicine, Northwestern University, Chicago, Illinois; ** Division of Infectious Diseases, Department of Medicine, University of Utah School of Medicine, Salt Lake City, Utah

**Keywords:** COVID-19, athlete, obesity, diabetes, asthma

Young athletes, generally speaking, represent one of the healthiest and fittest groups in society. The resumption of sport and exercise will ultimately bring athletes into closer proximity than is recommended by current physical distancing practices, potentially increasing one’s risk of infection. Data are emerging that high-intensity exercise may increase aerosolization and transmissibility of severe acute respiratory syndrome coronavirus-2 (SARS-CoV-2) from presymptomatic and mildly symptomatic individuals.^9^ It is in this setting that medical providers have wondered how to best counsel athletes with medical conditions that may be associated with increased risk of severe coronavirus disease (COVID-19), specifically, diabetes, asthma, sickle cell trait (SCT), hypertension, and obesity. The Centers for Disease Control and Prevention (CDC) guidance does not explicitly address young athletes returning to sport but recommends that high-risk individuals of any age take extra precautions, including those with chronic lung disease or moderate to severe asthma, chronic kidney disease being treated with dialysis, diabetes mellitus, hemoglobin disorders, liver disease, serious heart conditions, severe obesity (body mass index [BMI] ≥40 kg/m^2^), or who are immunocompromised.^2^ There is limited epidemiologic evidence to inform guidance for such individuals, especially in the high school and college age groups; however, young people in general are at low risk for poor outcomes including death, hospitalization, and severe disease. Here, we offer our appraisal of the situation and provide information that may help frame discussions with athletes.

The vast majority of deaths from coronavirus occur in those older than 25 years of age, with those aged 15 to 24 years representing only 0.1% of all deaths.^3^ Of those younger than 24 years of age who died from the virus, 4% had hypertension, 21% were obese, and 15% had diabetes.^3^ Prevalence of these conditions in the general adolescent population is similar for hypertension (4%)^8^ and obesity (20.6%)^6^ but is much lower for diabetes (0.25%).^1^ While no conclusions can be drawn based on limited data, the outcomes in this age group are consistent with the poor outcomes seen in patients with diabetes who contract COVID-19 in the general population; 1 study showed a 4-fold increased risk of severe/critical illness independent of other risk factors.^16^ Athletes with diabetes should be counseled regarding the potential for increased morbidity and mortality if infected with COVID-19 and consider delaying return until sports reintegration is confirmed safe and the risk of acquiring a new infection is better understood.

Asthma affects 8.4% of the population from 0 to 17 years of age with about 5% experiencing 1 or more asthma attacks per year.^5^ CDC guidelines suggest that only those with moderate to severe asthma are in the high-risk category, while mild, well-controlled asthma is not considered a risk for poor outcomes.^12^ Moderate asthma is characterized as having daily symptoms that cause some limitation of normal activities and a forced expiratory volume (FEV) of 60% to 80%.^12^ Exercise-induced bronchospasm is common among athletes, especially during the winter and in endurance sports, but is not known to confer a higher risk of poor outcomes with SARS-CoV-2 infection. Athletes with asthma should be evaluated prior to participation in sports to confirm their treatment regimen is optimized and they are adherent to their medications. Additionally, they should be advised to notify their medical team of any exacerbations of existing medical conditions or illness. Participation should be avoided in any individual with worsening asthma control.

SCT is also common, with 9% of African American/black individuals carrying the gene. Although generally considered benign, SCT has been associated with an increased risk of exertional death in football athletes and military recruits, as well as increased risk of venous thromboembolism.^7,13^ Although sickle cell disease and thalassemia are considered by the CDC as higher risk for adverse outcomes with COVID-19 infection, SCT is not. No additional precautions are recommended for returning athletes with SCT; however, if an athlete with SCT contracts SARS-CoV-2, treating physicians should be vigilant for issues related to hypercoagulability both during the acute illness and for several months into recovery. This includes allowing adequate acclimatization and reconditioning while optimizing hydration, minimizing heat stress, and avoiding blood flow restriction devices used for rehabilitation and strengthening.

The prevalence of hypertension in athletes appears to be similar to that of the general adolescent population (4%); however, studies suggest that American football lineman with higher BMI are more likely to be affected.^4^ Studies of hypertension in athletes are limited and often rely on one-time blood pressure measurements rather than standard diagnostic criteria. The frequency with which COVID-19 patients are hypertensive is not unexpected, given the age distribution of SARS-CoV-2 infection, and does not necessarily imply a causal relationship. However, concern remains that hypertension may be an independent risk factor for poor outcomes even though the CDC does not currently list hypertension as a predictor of severe illness.^2,15^ There was initial concern that treatment of hypertension with angiotensin-converting enzyme (ACE) inhibitors or angiotensin receptor blockers (ARBs) could increase the susceptibility to COVID-19 infection or worsen its course; however, several large studies did not support these hypotheses, and the American Heart Association does not recommend stopping ACE inhibitors or ARBs in patients with COVID-19.^11,14^ Thus, athletes with controlled hypertension should maintain their current regimen when returning to participation. Newly diagnosed hypertension should be treated as clinically appropriate. Preparticipation screening should include accurate measurement of blood pressure with repeat measurements (if abnormal) and presenting an opportunity to engage the athlete in discussions regarding treatment.

Finally, although most athletes are fit, many sports recruit athletes with a larger build, particularly American football lineman. The CDC groups people with severe obesity (BMI≥40 kg/m^2^) as potentially at risk for severe illness, although the literature associated with COVID-19 employs variable definitions of obesity, some including those with BMI ≥25 kg/m^2^.^2^ BMI is intended to be a marker of excess fat but may not be a good measure in athletes, as lean muscle mass is typically increased with lower percentages of body fat. Studies have shown an increased association of poor COVID-19 outcomes with higher BMIs. One study found this to be true especially in younger patients with BMIs ≥30 kg/m^2^ and even more so among those with BMI ≥35 kg/m^2^.^10^ As in the general population, those with higher BMI are also more likely to have other comorbidities such as hypertension or diabetes. Athletes with a high BMI, particularly those with a higher percentage of body fat, should be counseled on the overall adverse health effects of obesity and potential association with complications from COVID-19 and should be supported in adopting a healthy lifestyle. Lifestyle modifications in athletes who derive competitive advantage from higher BMI and are unwilling to consider weight reduction may be limited but should be addressed.

Preparticipation guidance for all athletes should include a discussion of risks of SARS-CoV-2. Athletes with diabetes appear to be at higher risk for poor outcomes; however, there is no evidence that athletes with asthma, SCT, and hypertension are at higher risk of poor outcomes, and it is unclear if obese athletes are at higher risk. All athletes should be engaged in a shared decision-making process that involves education, acknowledgement of uncertainty, and optimization of medical treatment. The risk of poor outcomes in those younger than 25 years of age remains low, and youth and high levels of fitness may effectively mitigate the risk of severe COVID-19 outcomes in athletes with pre-existing risk factors. The collection of these data is critical to better understanding the interplay of these elements. Exercise and sport are beneficial for both physical and mental health. We should attempt to mitigate risks for returning athletes by addressing potential risk factors, encouraging compliance with public health guidelines, and adjusting recommendations as the situation and evidence evolve.
